# Antiseptic Action of Acriflavine, Proflavine and Brilliant Green

**Published:** 1918-08

**Authors:** 


**Antiseptic Action of Acriflavine, Proflavine and Brilliant Green.** Gulbransen and Thornton, Brit. Med. Jour., states that the antiseptic action is slowly progressive, without interfering with phagocytosis or the welfare of the body cells generally. The flavine compounds may be safely used in the peritoneum and their action is enhanced by the presence of serum while brilliant green, like most other antiseptics has its action reduced by the presence of serum. The practical choice is obvious.

